# Supplementary material for: Comparative analysis of pre-Covid19 child immunization rates across 30 European countries and identification of underlying positive societal and system influences
Source: PLoS One. 2022 Aug 3;17(8):e0271290. doi: 10.1371/journal.pone.0271290 (PMC9348723; doi:10.1371/journal.pone.0271290)
Supplement: S1 File — (DOCX) [file pone.0271290.s001.docx]

# Supporting Information

### Decentralization

This is a categorical variable identifying the degree of centralization/decentralization of national health systems. It is coded based on a European Union analysis [29], and it takes five distinct values: centralized; mostly centralized; operatively centralized; partially decentralized; and decentralized. To estimate the effect of the different categories, in the regressions we chose to add them as separate dummy variables [30].

- 1. Centralized (i.e. all the power, responsibility and functions are with the central government or are deconcentrated, i.e. are given to entities at the territorial level which represent the central level).
  2. Mostly centralized (i.e. most of the power, responsibility and functions are with the central government, but lower levels of elected government still have a minor role in relation to health expenditure).
  3. Operatively decentralized (i.e. the central government has an important role within the health management system, but some operative functions are held by lower levels of the elected government).
  4. Partially decentralized (i.e. some of the power, responsibility and functions for health are transferred/devolved from the central government to lower, elected levels of government. The central government still has a role within the health management system, the importance of this role varying depending on the level of devolution).
  5. Decentralized (i.e. except for some main framing conditions, the power, responsibility, and functions for health are not with the central government but with lower, elected levels of government).

### Econometric strategy

To assess the effect of the different independent variables on vaccination coverage, we will estimate three different panel regressions (Equation 1), to be able to exploit the panel nature of data.

$$Y_{it}=\beta_{0}+\beta_{1}X_{it}+\beta_{2}X_{it}+\ldots+\beta_{k}X_{it}+ \mu$$

where y represents the dependent variable, $\beta_{0}$ the constant term, X represents the independent variables and $\mu$ represents the error term.

The three regressions are estimated upon different models in which each regression incorporates addition variables with respect to the previous one.

Substituting the terms with the variables employed in the analysis, the three equations become:

#### Equation 1

$${Average coverage}_{it}=\beta_{0}+\beta_{1}{GDP per capita}_{\mathrm{it}}+\beta_{2}{GINI index}_{it}+\beta_{3}{Tertiary education}_{it}+\beta_{4}{Child proportion}_{it}+\beta_{5}{Rural population}_{it} + \beta_{6}Country dummies+\mu$$

#### Equation 2

$${Average coverage}_{it}=\beta_{0}+\beta_{1}{GDP per capita}_{\mathrm{it}}+\beta_{2}{GINI index}_{it}+\beta_{3}{Tertiary education}_{it}+\beta_{4}{Child proportion}_{it}+\beta_{5}{Rural population}_{it}+\beta_{6}{Nurses/doctors ratio}_{it}+\beta_{7}{Decentralized}_{it}+\beta_{8}{Paediatrician lead}_{it}+\beta_{9}Country dummies+\mu$$

#### Equation 3

$${Average coverage}_{it}=\beta_{0}+\beta_{1}{GDP per capita}_{\mathrm{it}}+\beta_{2}{GINI index}_{it}+\beta_{3}{Tertiary education}_{it}+\beta_{4}{Child proportion}_{it}+\beta_{5}{Rural population}_{it}+\beta_{6}{Nurses/doctors ratio}_{it}+\beta_{7}{Decentralized}_{it}+\beta_{8}{Paediatrician lead}_{it}+\beta_{9}{Mandatory vaccination}_{it}+\beta_{10}{Child health strategy}_{it}+\beta_{11}{Child e-health strategy}_{it}+\beta_{12}{Home-based records}_{it} + \beta_{13}Country dummies+\mu$$

Equation 1, 2 and 3 are estimated by employing country dummies to control for the presence of country-specific factors potentially affecting vaccination coverage.

Table 1 reports the countries and years covered by the analysed sample.

Table 1. Countries involved in the analysis with relevant years where data was available

| **Number** | **Country** | **year** |
| --- | --- | --- |
| 1 | Austria | 2007-2017 |
| 2 | Belgium | 2004-2017 |
| 3 | Bulgaria | 1992-2015 |
| 4 | Croatia | 2001-2016 |
| 5 | Cyprus | 1991-2016 |
| 6 | Czech Republic | 2000-2017 |
| 7 | Estonia | 2000-2017 |
| 8 | France | 2000-2017 |
| 9 | Germany | 2013-2017 |
| 10 | Greece | 1999-2017 |
| 11 | Hungary | 2000-2017 |
| 12 | Ireland | 2010-2017 |
| 13 | Italy | 2003-2017 |
| 14 | Latvia | 1999-2017 |
| 15 | Lithuania | 1998-2017 |
| 16 | Luxembourg | 2006-2017 |
| 17 | Malta | 2008-2015 |
| 18 | Netherlands | 2015-2017 |
| 19 | Poland | 1995-2017 |
| 20 | Portugal | 2002-2017 |
| 21 | Romania | 2000-2017 |
| 22 | Slovakia | 2000-2016 |
| 23 | Slovenia | 2000-2004 |
| 24 | Spain | 1996-2017 |
| 25 | Sweden | 2005-2016 |
