# Supplementary material for: Comparative analysis of pre-Covid19 child immunization rates across 30 European countries and identification of underlying positive societal and system influences
Source: PLoS One. 2022 Aug 3;17(8):e0271290. doi: 10.1371/journal.pone.0271290 (PMC9348723; doi:10.1371/journal.pone.0271290)
Supplement: Attachment — Submitted filename: Response to the Editor Paper vaccination_PLOS.docx [file pone.0271290.s002.docx]

Dear Editor,

We thank you for your email regarding our submission to the PLOS one journal. We are grateful for your suggestions and the comments.

We have answered the comments raised in a point-by-point format addressing any concerns. Comments are reported in red in the following. The manuscript has considerably improved by these modifications. We have highlighted the changes in the main text.

We sincerely hope that these revisions will speed up the review process considering that we have submitted this paper on October 2021, more than five months ago.

Best regards,

Fabrizio Pecoraro

**Journal requirements:**

1. Details should be provided on when data on each country characteristics (such as policies) have been extracted. They may vary and we need to know when the assessment was made.

A specific table (Table 1) has been included in the main text of the manuscript to report detailed information on data typology, unit of measure, years of coverage, source and date of access of each characteristic.

1. The description of the Tables is poor overall. There are many crucial details that have not been explained and must be explained. E.g. in Table 1, what is CoV? "Antigen" should be replace by "Vaccine", in Table 2 what 368 observations stands for? No units of measures are given, 3 decimals are not needed, min./max. can be cut, in Table 3, there is no explanation of what is the dependent variable, the title average coverage is wrongly placed, in place of "covariates" or "variables", six decimals are surreal (change the unit of the variable in thoushands), there is no indication that the numbers are referred to beta coefficients, the building of the final model creates confusion (are we searching the best model to evaluated an association, or are we making a statistical exercise?). All the figures do not have clear explanations and, as they currently stand, they are impossible to understand.

Captions and table descriptions have been improved to better describe tables and figures. As you suggested, we included the unit of measure of each variable and changed the number of digits accordingly.

2. Why non significant variables have been retained in the final model has to be explained, and once the final, most complete model has been fit, there is no need at all to leave the results of the intermediate models, unless there are very major reasons. As it currently stands, although the aim is straightforward, it is confusing and very difficult to understand. Again, in the results there is a long description of the three models, which is most likely trivial. Please discuss the results of the best model.

As you suggested we excluded the first and the second models from the manuscript leaving only the final regression.

Overall, the writing is fair, but can be improved. The topic is complex, and the authors should try to make the sentences as short as possible. As it currently stands, it is sometimes too complex for the reader to understand the concepts. Some sentences are simply impossible to understand and must be rewritten. E.g. "The inter-country analysis showed that, besides a small set of high-performing countries, the level of variability can be differently associated with countries showing vaccination coverage rates that meet the target of 95% only with some vaccines, or with countries showing low uptake rates related to almost all vaccines". Other sentences are written in Italian and then translated, but must be improved. E.g. "gives much greater richness of analysis and creates some stable and important findings" or "Though perforce based on snapshot national data". Overall, the writing of manuscript should be revised in depth, keeping in mind that the discussion is very long and only truly essential concepts should be maintained.

The text has been improved.

3. Please address errors such as "Error! Reference source not found"

We are sorry for this mistake. It seems that the conversion from docx to pdf remove the table and figure references replacing them with this sentence. We will pay more attention in this submission.

After these issues are addressed, the manuscript will proceed to the review process. The study is valid.
