# Supplementary material for: Comparative analysis of pre-Covid19 child immunization rates across 30 European countries and identification of underlying positive societal and system influences
Source: PLoS One. 2022 Aug 3;17(8):e0271290. doi: 10.1371/journal.pone.0271290 (PMC9348723; doi:10.1371/journal.pone.0271290)
Supplement: Attachment — Submitted filename: Answers to the Reviewers_d.docx [file pone.0271290.s003.docx]

**Answers to the Reviewers**

We would firstly like to thank the Editor and the Reviewer for their useful, pertinent, and insightful comments and suggestions. The issues raised by the Reviewer have been addressed throughout this new version of the paper.

Following the detailed responses to the issues raised by the two Reviewer.

*“For example, the authors found that a high nurse/doctor ratio had a positive effect and argue that this may result in healthcare being more holistic, reflecting person centred values and focussed away from purely biomedical interventions. They acknowledge that the data on nurse/doctor ratio does not allow the extent to which nurses are employed in preventive care or vaccination activities and recommend better information is needed in this respect. This is a fundamental question - could this conclusion lead to countries deciding to employ fewer doctors?!. However, an important issues is that in many countries, vaccination is doctor led rather than nurse led - this information should be easily available.”*

The employment of the nurses/doctor ratio was based on the willingness to understand whether a higher number of nurses compared to the number of doctors was associated with higher vaccination rates within countries. Indeed, this was based on the implicit assumption that nurses concur in vaccination, and more broadly preventive care, activities. Unfortunately, we do not know whether this is the case or not, since there is not available information on the role performed by nurses within the different health systems considered in the analysis. For these reasons, in this new version we further clarified and made more explicit that the results concerning this variable are only preliminary and that there is the need to perform further research to understand the different roles in vaccination activities that nurses perform in the different countries (page 6 line 119; page 20 line 383).

*However, the issue over which I have most comment is the conclusion that mandatory vaccination has a negative relationship with vaccination coverage. Key to this finding is how long the mandatory vaccination policy has been in place. In some of the countries included in this analysis, mandatory vaccination was only introduced or requirements expanded in 2017/2018, the year taken in this project as the ‘anchor year’, in response to wide spread measles outbreaks, giving no time for the effects of mandation. The lower vaccine uptake in these countries may thus have resulted in the introduction of mandation and is not the cause of the low uptake.*

We coded the “mandatory” variable based on the work of Bozzola et al. (2010). In particular, we coded it as a dummy variable taking the value of 0 (in all the years analysed) if no mandatory vaccination has ever been in place in a certain country and taking value of 1 otherwise. This choice was dictated by the fact that we employed the average value of several vaccine’s uptake rather than focusing on a single vaccine and that our interests was to test whether the presence of mandatory vaccinations within a country was associated with higher or lower rates. In other words, we did not assess the lag between the policy implementation and the changes in vaccination rates rather the direction of the relation between the presence of at least one mandatory vaccine, at aggregated level, and the average vaccination rates. *Overall, I am not clear about the take home message from this paper other than richer countries, with cohesive management structures have higher vaccination rates. How would a country wishing to improve its vaccination uptake act on these findings? The study is an impressive use of data, but I do not feel takes us much further in understanding the determinants of vaccine uptake.*

Following your comment, in this new version of the paper we added a paragraph explicitly describing the implication of our results in terms of policy (page 22 line 452). *There are a number of minor details which require explanation for an international readership or correction:
Child e-health strategy, home based records – are both defined, but would benefits from further explanation. For example, in UK, home based records are known as Personal Child Health Records.
Page 16, line 331 – presume this should be ‘immunisation’ data not ‘immunological’*

We clarified the meaning of Child health strategy and home-based records. We also did a proof reading of the entire document.
